# Recombinant protein of *Haemonchus contortus* small GTPase ADP-ribosylation factor 1 (HcARF1) modulate the cell mediated immune response *in vitro*

**DOI:** 10.18632/oncotarget.22662

**Published:** 2017-11-26

**Authors:** Javaid Ali Gadahi, Muhammad Ehsan, Shuai Wang, Zhenchao Zhang, Ruofeng Yan, Xiaokai Song, Lixin Xu, Xiangrui Li

**Affiliations:** ^1^ College of Veterinary Medicine, Nanjing Agricultural University, Nanjing, PR China; ^2^ Department of Veterinary Parasitology, Sindh Agriculture University, Tando Jam, Pakistan

**Keywords:** H. contortus, ADP-ribosylation factor, PBMCs, cytokines, proliferation

## Abstract

ADP-ribosylation factors (ARFs) are members of the Ras-related small GTPase family involved in the vesicular trafficking regulation. Immunomodulatory effects of these proteinson host cell arenot being addressed yet. *H. contortus* small GTPase ADP-ribosylation 1 gene (HcARF1) was cloned and recombinant protein of HcARF1 (rHcARF1) was successfully expressed in *Escherichia coli*. Binding activity of rHcARF1 to goat PBMCs was confirmed by immunofluorescence assay (IFA) and its immunomudulatory effects on cytokine secretion, cell proliferation, cell migration and nitric oxide production (NO) were observed by co-incubation of rHcARF1. IFA results revealed that rHcARF1 could bind to the PBMCs. The interaction of rHcARF1 modulated the cytokine production, the production of IL-4, IL-10 and IL-17 was increased in a dose dependent manner, however, the IFN-γ production was significantly decreased. Cell migration and NO production were significantly increased by rHcARF1, whereas, rHcARF1 treatment significantly suppressed the proliferation of the PBMC in a dose dependent manner. Our findings showed that the rHcARF1 play important roles on the goat PBMCs.

## INTRODUCTION

ADP-ribosylation factors (ARFs) are member of the Ras-related small GTPases family also known as low molecular weight guanine-nucleotide-binding (G) proteins [1] and their involvement in the vesicular trafficking regulation has been well characterized [2]. ARF1 is characteristically related with the golgi and in some cell types also be found related with the plasma membrane and is an important regulator of the biological process induced by epidermal growth factor [3–7]. Involvement of the ARF1 in the activation of signaling molecules, such as phospholipase D, PI3K and type I phosphatidylinositol 4-phosphate 5-kinase has been reported, these data suggests that in addition of trafficking regulation, ARF1 GTPase also act as a signal transducer [5, 8–10].

ARF1 is involved in membrane affinity and it actively involve in the formation of non-clatherin/clatherin coated vesicles which helps in the transportation of vesicles to carry important cellular components required for biological processes such as cell signaling [11]. ARF1 also involved in the activation of PLD enzyme which cleaves phophatidylcholine to generate phophatidic acid (PA) and choline. PA could modulate many cellular events like DNA synthesis, cell proliferation, and secretory responses [12]. Characterization of the ARF proteins has been performed in various parasites included *Caenorhabditis elegans* [13, 14]*, Entamoeba histolytica* [15], *Plasmodium falciparum* [16, 17] and *Leishmania* [18].

*Haemonchus contortus* is an abomasal nematode parasite, it is the most important parasitic problem on a global basis [19]. *H. contortus* is responsible for the decline of rural economy due to weight loss and anemia resulted in decreased meat and milk production and *H. contortus* is one of the comprehensively used parasitic nematode for drug discovery, vaccine development and drug resistance [20–23]. The animal's body triggers several defense mechanisms during *H. contortus* infection to control the infection in different ways, such as reactive oxygen species production by immune cells. This protection may leads to damages of various host cells and tissues by oxidative stress [24, 25].

Previously, we identified that ARF1 protein was one of the interacting protein with goat PBMCs at multiple developmental stages *in vivo* [26]. Molecular cloning and functional characterization of *H. contortus* ARF1 has not being addressed yet. In the current study, recombinant protein of HcARF1 (rHcARF1) was constructed and its immunomodulatory effects on the goat PBMCs was evaluated.

## RESULTS

### Sequence and phylogenetic analysis

**Figure 1:**
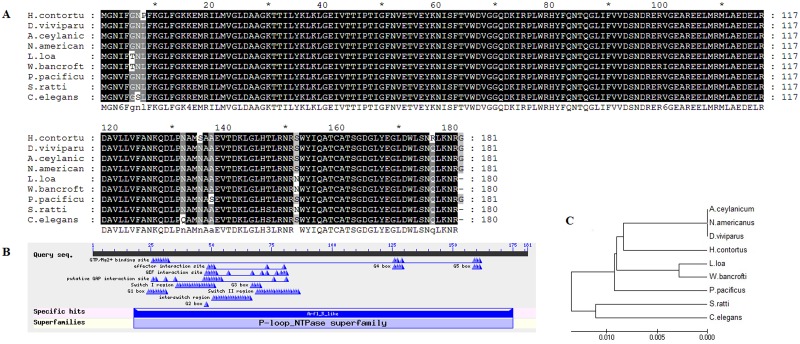
Multiple alignment of amino acid sequence of HcARF1 **A.** The amino acid sequence of HcARF1 aligned with other organism ARF family reported in the NCBI database of *Ancylostoma ceylanicum* (98%), *Dictyocaulus viviparous* (98%), *Necator americanus* (98%), *Loa loa* (97%), S*trongyloides ratti* (97%), *Wuchereria bancrofti* (97%), *Pristionchus pacificus* (97%), *Caenorhabditis elegans* (96%). **B.** Putative conserved domain. **C.** Phylogenetic analysis of the relationships among the amino acid sequences of *HcARF1* and known similar sequences by minimum evolution.

The recombinant plasmid pET32a-HcARF1 was confirmed by restriction enzyme digestion and sequencing. The results of the BLASTx revealed that, ORF contains 546 bp encodes 181 amino acids. The deduced protein sequence of HcARF1 was used for multiple sequence alignment (Figure 1A). The results of the multiple alignments showed that HcARF1 is very close to the ADP-ribosylation factor family protein of *Ancylostoma ceylanicum* (98%), *Dictyocaulus viviparous* (98%), *Necator americanus* (98%), *Loa loa* (97%), S*trongyloides ratti* (97%), *Wuchereria bancrofti* (97%), *Pristionchus pacificus* (97%) *Caenorhabditis elegans* (96%), The typical characteristics of the HcARF1 were confirmed as ARF 1-5 by their GTP/Mg2 binding and putative GAP interaction sites (Figure 1B). These findings confirmed that, the cloned ORF belongs to the *H. contortus* ARF1 family. The phylogenic tree analysis indicated that *HcARF1* was closely related to ARF of homologous protein sequence obtained from NCBI data base (Figure 1C).

### Expression and purification of rHcARF1

**Figure 2:**
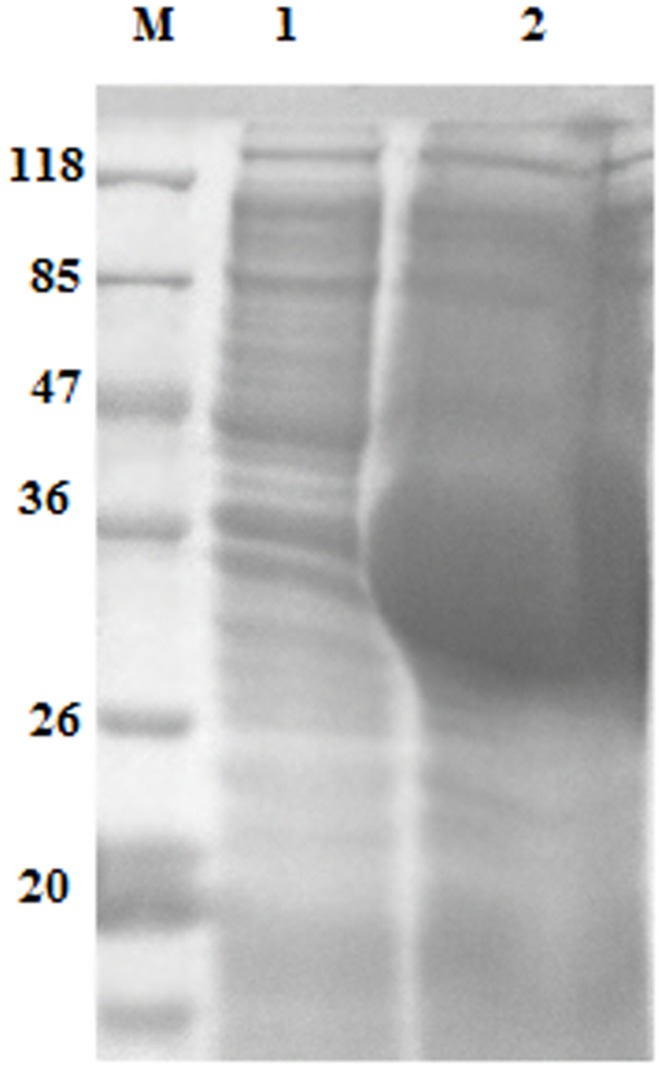
Expression of rHcARF1 protein after induction with 1mM IPTG Lane M: standard protein molecular weight marker, 1: recombinant expression vector before induction, Lane 2 expression after induction.

The recombinant protein of HcARF1 was expressed and purified as a His tagged fusion protein. The expressed protein was detected at 38 kDa, it is higher than the calculated mass of 20 kDa of HcARF1 because of extra molecular mass of pET 32a expression vector (Figure 2).

### Detection of recombinant HcARF1 protein by immunoblotting

**Figure 3:**
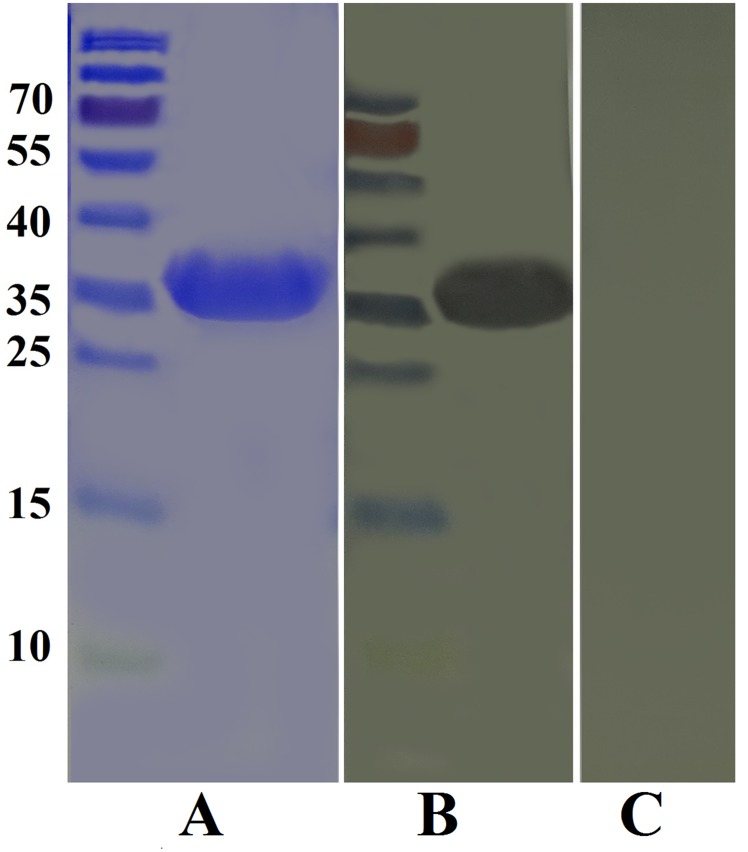
Western blot analysis of rHcARF1 **A.** Purified rHcARF1 was electrophoresed in SDS-PAGE and stained with Coomassie blue, **B.** then transferred to a membrane for western blot analysis with rat anti-rHcARF1 sera and **C.** normal rat sera as control.

The results of the immunoblot indicated that the rHcARF1 was detected by rat anti rHcARF1, but in negative control no protein was identified by normal rat sera (Figure 3).

### Interaction of rHcARF1 with goat PBMCs

**Figure 4:**
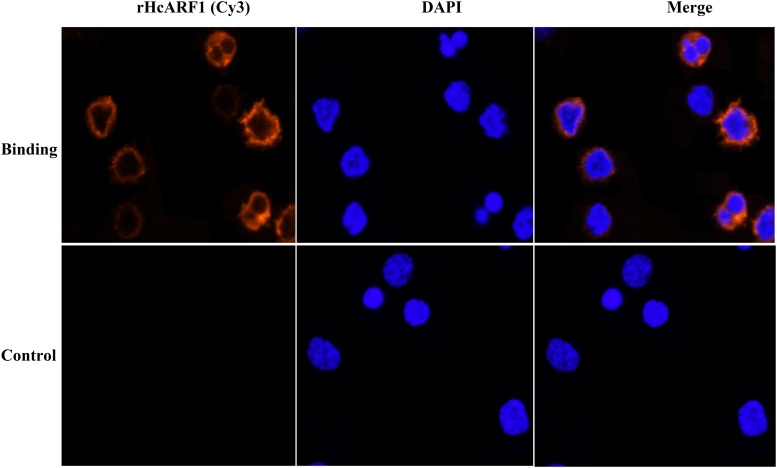
Confirmation of binding of rHcARF1 to goat PBMCs by IFA The nuclei of the corresponding cells were visualized by DAPI (blue) staining. Staining of the target proteins (red) were visualized by Cy3-conjugated secondary antibody. Merge, overlap of red and blue channels. No red fluorescence was observed in control group.

Immunofluorescence assay (IFA) was used to confirm the interaction of rHcARF1 with host PBMCs. Confocal microscopy indicated that rHcARF1 was interacted with the cell surface (red fluorescence). In the control group, no binding was observed (Figure 4).

### The binding of rHcARF1 to goat PMBCs increased IL-4, IL-10 and IL-17 and suppressed IFN-γ

**Figure 5:**
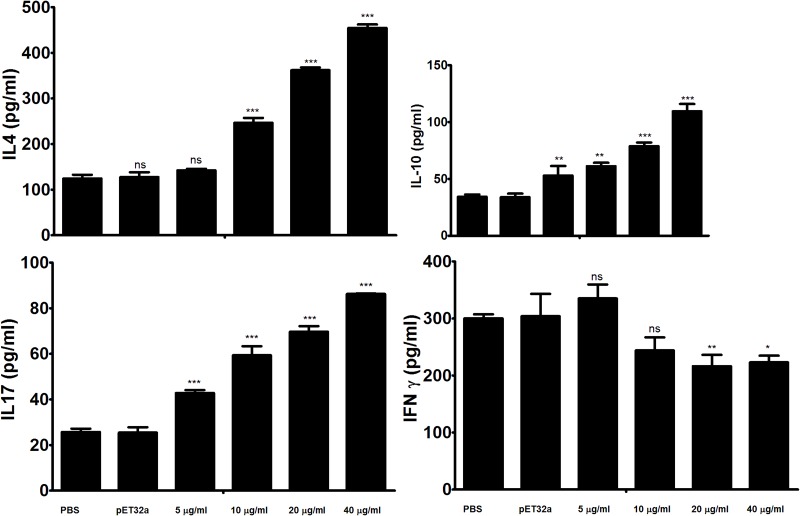
Analysis of the level of multiple cytokine production by PBMCs *in vitro* PBMCs were stimulated with ConA (10 μg/ml) for 24 h in the presence or absence of various concentrations of rHcARF1 and pET32a. Cytokine secretion in the supernatant of cell cultures was quantified by ELISA. The data are representative of three independent experiments (^*^p <0.01, ^**^p < 0.001, ns non significant).

In the present study ELISA was used to analyze the impacts of the rHcARF1 on the cytokine production. Our findings indicated that rHcARF1 modulating the cytokine production (Figure 5). Secretion of IL-4, IL-10 and IL-17 was significantly increased whereas, the production of the IFN-γ was decreased in PBMCs incubated with different concentration of rHcARF1.

Interaction of rHcARF1 with goat PBMCs significantly increased the production of cytokine IL-4, IL-10 and IL-17 in a dose dependent manner. On the contrary, type II interferon (IFN-γ) was decreased by the interaction of rHcARF1 (Figure 5).

### The interaction of rHcARF1 with goat PMBCs increased cell migration

**Figure 6:**
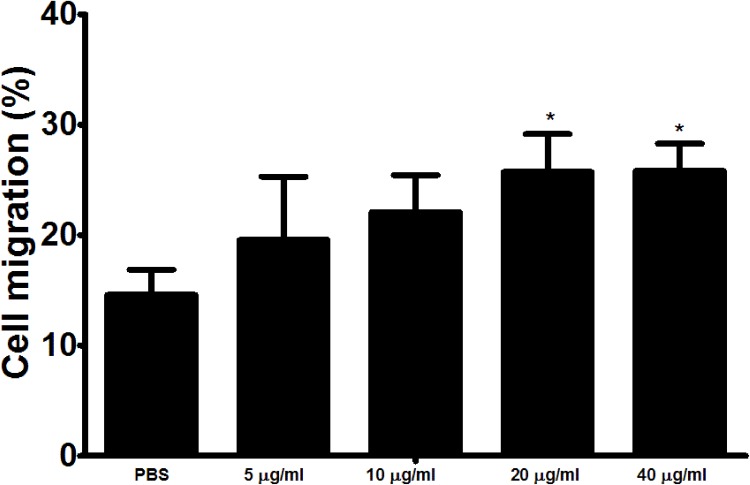
Impact of the various concentration of rHcARF1 on PBMC migration PBMC were treated with control buffer and different concentrations of rHcARF1, Then the random migration was determined. The difference between the mean values was calculated using ANOVA. Data are representative of 3 independent experiments; ^*^p < 0.01versus the control.

In the current study, cell migration assay was performed to appraise the effect of rHcARF1 on cell migration (Figure 6). Our findings showed that cell migration was significantly increased in cells incubated with 20 and 40 μg/ml of rHcARF1.

### The interaction of rHcARF1 with goat PMBCs decreased cell proliferation

**Figure 7:**
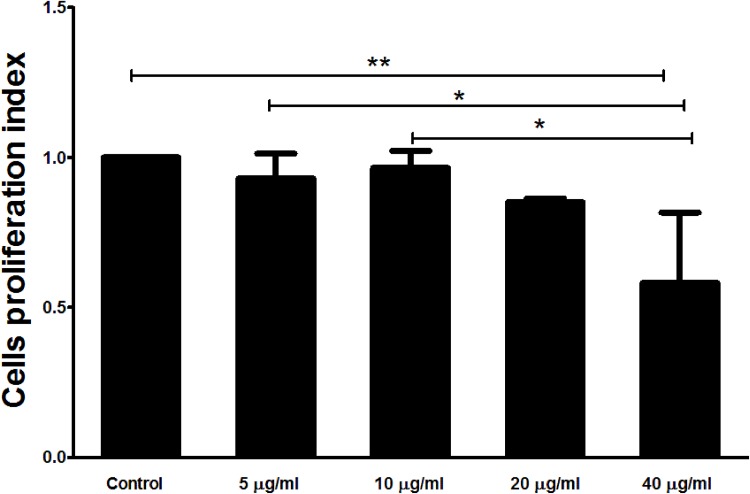
Effects of rHcARF1 on PBMCs proliferation Cells were activated with ConA and incubated at the same time with serial concentrations of rHcARF1 at 37°C and 5% CO_2_. The proliferation was measured by CCK-8 incorporation after 72 h. Cell proliferation index was calculated considering the OD_450_ values in controls as 100%. The data were representative of three independent experiments (^*^p < 0.01 and ^**^p < 0.001).

The treatment of rHcARF1 significantly decreased the multiplication of the PBMC at the concentration 40 μg/ml as compared to the control group (Figure 7).

### The binding of rHcARF1 to goat PMBCs increased nitric oxide production

**Figure 8:**
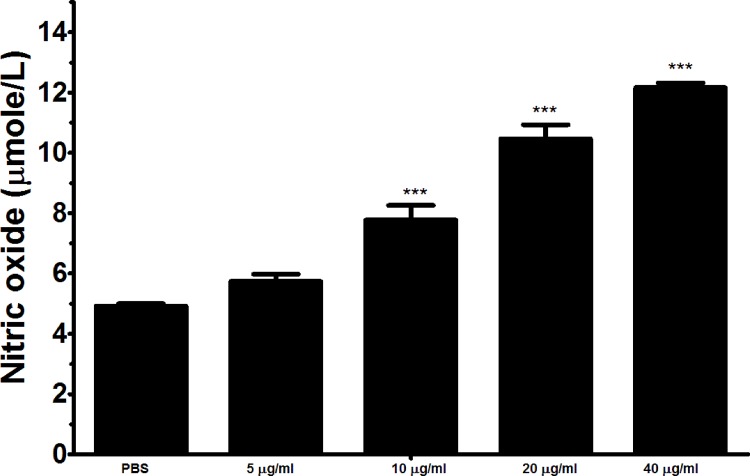
Effects of rHARF1 on nitric oxide production by PBMCs *in vitro* Cells were activated with ConA and incubated at the same time with serial concentrations of rHcARF1 at 37°C and 5% CO_2_. The nitrite concentration in the PBMCs was measured by using the Griess assay and used as an indicator of nitric oxide production by the PBMCs. The data were representative of three independent experiments (^***^p < 0.0001).

Total nitric oxide assay kit was used to measure the Nitric oxide (NO) production by PBMCs incubated with various concentration of rHcARF1. Our findings showed that, rHcARF1 significantly increased the NO production at 10, 20 and 40μg/ml (Figure 8).

## DISCUSSION

A GTP-binding protein has been concerned in the regulation of membrane traffic by the secretory pathway and ARFs are highly conserved family of eukaryotic small GTP-binding proteins with roles in membrane dynamics and vesicle trafficking [27, 28]. ARFs proteins also being identified in different parasites [13, 15, 29–32].

To date, little information is available on the *ARFs* in the parasitic nematode, in case of *H. contortus* molecular and functional characterization has not been done. In the current research we have cloned a small GTPase ARF1 gene from the *Haemonchus contortus* cDNA. The multiple sequence alignment of HcARF1 with other organism indicated that the phylogenetic relationship of *H. contortus* was closely related with the nematodes (*Ancylostoma ceylanicum, Angiostrongylus cantonensis, Dictyocaulus viviparous, Dirofilaria immitis and Loa loa*)*.* The multiple sequence alignment of HcARF1 indicated that cloned gene belongs to the ARF protein family.

Cytokine produced by the immune cells are responsible for communication as well as regulation of the immune system. First line of the defense against various organisms including parasites provided by the innate immune system by toll-like receptors (TLRs) family of pattern recognition receptors (PRRs) that recognize the broad range of pathogen-associated molecular patterns (PAMPs) [33, 34].

In the present study, rHcARF1 increased cytokines IL-4, IL-10 and IL-17 secretion by PBMCs but decreased the secretion of Type II interferon (IFN-γ). Previously, it was suggested that an ADP ribosylation factor-GTPase activating protein 1 was negatively regulated LPS-induced pro-inflammatory mediators production by down-regulation of LPS signaling [35]. Wu et. al reported that, class III PI3K/ARF6-dependent pathway was involved in regulating cellular subsequently modulates CpGODN/TLR9-signaling cascades such as TLR9 trafficking and cytokine production [36]. In our study, cytokine secretion modulated by rHcARF1 strongly indicated the multiple and distinct regulatory effects of ARF on goat immune cells.

It is generally considered that, type 2 immunity (Th2) associated with secretion of IL-4 and IL-5 is the main immune mechanism against helminths including *H. contortus* [37–39]. In our previous study we described that interaction of HcESPs with goat PBMCs decreased the production of IL-4 *in vitro* [40]. The findings of our study conflicting the above results. Here, we found that the rHcARF1 could increase the production of IL-4. These results directed that rHcARF1 might be played a part in the initiation of the Th2 immunity. However, HcESPs are the group of various proteins, thus proteins involved in decline of IL-4 are worthy of further investigations.

IL-17 a cytokine is strong inducer of inflammation produced by Th17 cells [41] and it is concerned with pathogenesis [42–48]. In our previous studies we found that HcARF1 could interact with host PBMCs at various developmental stages of *H. contortus* [26] and the secretion of IL-17 was increased by the interaction of HcESPs [40]. Here, we reported that rHcARF1 enhanced the production of IL-17, which indicated that rHcARF1 could part play some roles in the HcESPs on IL-17 production.

It has been recognized that immune suppressive cytokine IL-10 secreted by inducible Treg cells (iT_Reg_) suppress the IFN-γ production [49, 50]. The generation of immune suppressive cytokine IL-10 might be an important tactic by which parasites could suppress the IFN-γ-dependent, cell-mediated immunity [51, 52]. In our previous research we found that rHcESPs suppressed the immune response by increasing the IL-10 and decreasing IFN-γ production [40]. Here we found that interaction of rHcARF1 with goat PBMCs could increase the IL-10 and decrease the IFN-γ production. Therefore, we suggested that, rHcARF1 is very important protein of HcESPs that could suppress the Th1 immune response and might be beneficial for *H. contortus* evading from the host immunity at early stage.

Previous studies demonstrated that ARF could be regulating the cell cycle progression by the suppression of transcription factor E2F1 activity [53, 54]. Currently, the effects of the rHcARF1 on the cell proliferation was evaluated and results indicated that rHcARF1 significantly inhibited the cell proliferation. Complex regulatory activities was ultimately linked with each other, such as cell activation, cytokine secretion and cell cycling leading to cell proliferation [55].

NO has been reported as an important immune-mediator and play important role in immunoregulation in various infections including *H. contortus* by mediating host protection by parasite killing or by suppressing the growth [56–58]. Previously reported that, endogenous IL-17 was involved in T cell-mediated NO production [59, 60]. In the present study immune-modulating effects of the rHcARF1 on the NO production by goat PBMCs was evaluated. Cells incubated with rHcARF1 significantly increased the NO production in a dose dependent manner. This up-regulation might be associated with the increased level of IL-17. Our results indicated that, up-regulation of IL-17 as well as NO production might involve in the TH17/NO inflammatory response and pathogenesis.

In Conclusion, we firstly cloned the HcARF1 and demonstrated that HcARF1 is one of the active protein of HcESPs that might be involved in the immune modulation. These findings indicated that, the interaction of rHcARF1 with host cells increased the production of IL-4, IL-10, IL-17, NO and cell migration. However, the secretion of IFN-γ and proliferation of PBMCs was significantly decreased. Our results might help to understand the mechanism involved in host parasite interaction. The immune system has different cell populations including T and B lymphocytes, macrophages, antigen presenting cells, NK cells, etc. How does HcARF1 activate immune and cellular response and which immune cells have been actively involved during the infection need to be further researched.

## MATERIALS AND METHODS

### Ethics statement

Animal experiments were conducted following the guidelines of the Animal Ethics Committee, Nanjing Agricultural University, China. All experimental protocols were approved by the Science and Technology Agency of Jiangsu Province. The approval ID is SYXK (SU) 2010-0005.

### Synthesis of *Haemonchus contortus* cDNA

Total RNA was isolated from adult worms of *H. contortus* collected from the abomasums of donor goats as described previously [61]. The worms were ground using a pre-chilled mortor and pestle. One ml of Trizol (Invitrogen) was added and homogenized for 30 minutes. Then 200μl of Tri-chloromethane was added and the mixture was spun at 12,000 rpm for 15 min at 4°C. After that, RNA was precipitated from the supernatant by the addition of 0.25 volumes of isopropyl alcohol per each milliliter of Trizol and incubated at -20°C for 30 min. The RNA was pelleted at 12,000 rpm at 4°C for 10 min. Hereafter, RNA pellets were washed by 70% ethanol and dried. The Pellets were resuspended in DEPC-treated water and the RNA solution was used in subsequent cDNA preparation immediately. The cDNA was synthesized by reverse transcription reaction using cDNA Kit (TaKaRa Biotechnology) according to the manufacturer's instructions.

### Molecular cloning of HcARF1 and expression of recombinant HcARF1 protein (rHcARF1)

The complete open reading frame (ORF) of HcARF1 was amplified by reverse transcription-polymerase chain reaction (RT-PCR) using the designed primers of *H. contortus* ARF gene (GI: 533372025), gene bank accession number HF964523.1. The sense and antisense primer sequences are as the following: 5’- *GGATCC*ATGGGTAACATTTTCGG -3’ and 5’- *CTCGAG*TTATCCTCTGTTTTTCA -3.

The PCR products were purified by using E.Z.N.A. Gel Extraction Kit (Omega bio-tech, USA) and ligated into pMD19-T cloning vector (TaKaRa Biotechnology, China) and then transformed into *E. coli* DH_5α_ strain. The positive clones were confirmed by double digestion with *BamHI/Xho1* enzymes, and the selected positive recombinant clones were sequenced by Invitrogen Bio-tech (Shanghai, China). The sequence data was assembled and analyzed by DNAssist software version 2.2. The HcARF1 gene was then cloned into *BamHI/Xho1* sites of expression plasmid pET32a (+) vector (Novagen, USA). The recombinant plasmid was sequenced to confirm the correct insertion of HcARF1 gene in the proper reading frame.

The expression of the recombinant fusion protein in *E. coli* BL-2 1 cells (DE3) was induced by isopropy- ß D -thiogala ctoside (IPTG ) at a final concentration of 1mM for 4 h at 37°C in Luria-Bertini (LB) medium with ampicillin (100 μg/ml). The histidine-tagged fusion protein was purified from the supernatant of bacterial lysates using the His·Bind^®^Resin Chromatography kit (Novagen) and dialyzed in phosphate buffered saline (PBS, pH 7.4) to remove imidazole. Endotoxins were removed from the recombinant proteins using ToxinEraser^TM^ Endotoxin Removal kit (GeneScript, USA). The purity and concentration of the purified rHcARF1 was analyzed by 12% sodium dodecyl sulfate polyacrylamide gelelectrophoresis (SDS- PAGE ) followed by Coomassie blue staining.

### Sequence alignments and phylogenetic analysis of HcARF1

Sequence similarity was assessed using protein-protein basic local alignment search tools BLASTp and BLASTx sequences (http://www.blast.ncbi.nlm.nih.gov/Blast.cgi). HcARF1 sequences were aligned using ClustalX 1.83 program (http://www.clustal.org/). The phylogenetic tree was constructed by aligning the amino acid sequences using the Neighbor-Joining method and plotted and visualized using the Molecular Evolutionary Genetics Analysis 5.1 program (http://www.megasoftware.net/).

### Generation of polyclonal antibodies

To generate polyclonal antibodies against rHcARF1 , 0.4 mg of rHcARF1 was mixed with Freund's complete adjuvant (1:1) and injected subcutaneously into 3 female Sprague Dawley (SD) rats [62, 63]. Rats received four doses of injection with the same proteins at 2-week intervals. Ten days after the last injection, the rats were anesthetized with diethyl ether, and sera containing specific anti-rHcARF1 antibodies were collected. The concentration of antibodies was determined by ELISA. The specific reactivity with rHcARF1 was confirmed by western blot analysis.

### Immuno-blot for the rHcARF1

Purified rHcARF1 were resolved by 10% SDS-PAGE and transferred to polyvinylidene difluoride (PVDF) Membrane (Millipore, USA). Non-specific binding was blocked by incubating the membranes in 5% skim milk in Tris-buffered saline containing 0.1% Tween-20 (TBST) for 1 h at room temperature. The membranes were then washed 5 times (5 min each) with TBST, followed by incubation with the primary antibodies (anti-rHcARF1) for 1 h at 37 °C (1:100 dilution in TBST). After washing 5 times with TBST, the membranes were incubated with HRP-conjugated rabbit anti-rat IgG (Sigma, USA) for 1 h at 37 °C (diluted 1:2000 in TBST). Finally, the bound antibody was detected using 3,3-diaminobenzidine tetra hydrochloride (DAB) kit (Boster Bio-technology) according to manufacturer's instructions.

### Binding of rHcARF1 to goat PBMC

Freshly isolated PBMCs were incubated in the presence and absence (control) of rHcARF1 (5μg/ml) for 1 h at 37°C. Confirmation of binding was determined by an immunofluorescence assay (IFA) as described by Yuan et al. [56]. Briefly, washed cells (10^5^ / ml) were fixed with 4% paraformaldehyde on a poly-L-lysine-coated glass slide. The cells were then treated with blocking solution (4% BSA in PBS) for 30 min to minimize background staining. After sequential incubation with rat anti-rHcARF1 IgG (1:100) for 2 h and a secondary antibody (1:300) coupled to the fluorescent dye Cy3 (Beyotime, Jiangsu, China) for 1 h, nuclear staining with 2-(4-amidinophenyl)-6-indole carbamidinedihydrochloride (DAPI, 1.5 μM; Sigma, MO, USA) was performed for 6 min. Then, protein localization was determined by observing the staining patterns with a 100× oil objective lens on a laser scanning confocal microscope (L SM710, Zeiss, Jena, Germany). Digital images were captured using the Zeiss microscope software package ZEN 2012 (Zeiss, Jena, Germany).

### Detection of the cytokine levels by ELISA of PBMCs treated with rHcARF1

The freshly isolated PBMCs were re-suspended to a final density of 5 × 10^6^ /ml in complete medium (RPMI 1640 supplemented with 100 U/ml penicillin, 100 μg/ml streptomycin,2 mM L-glutamine, 10% FCS). In the test groups, cells were treated with ConA (10μg/ml) and different concentration of the rHcARF1 (5, 10, 20, and 40μg/ml). The control groups were treated with ConA in equal volume of PBS or ConA and recombinant protein of empty pET32a. Then, the cells were seeded into 24-well plates (1ml/well) and cultured for 24h in 5% CO_2_ atmosphere at 37 °C. The plates were then centrifuged at 200 × g for 15 min and the supernatants were collected. The levels of IL-4, IL-10, IL-17 and IFN-γ in supernatants were determined using commercially available goat ELISA kits (Jian cheng Biotech, China). The cell viability was assessed by means of the trypan blue exclusion test before the incubation of PBMCs with rHcARF1. Three individual experiments were performed.

### Cell migration assay

The cell migration assay was performed using a Transwell system (Corning, USA), this allowed cells to migrate throughout an 8 μm pore size polycarbonate membrane [27]. The treatment group was incubated with different concentrations of rHcARF1 (5, 10, 20, and 40μg/ml) and the control group was treated with an equal volume of PBS. Each experiment was performed in triplicate.

### Cell proliferation assay

Cell proliferation assay was performed as previously described [64]. Briefly, 100 μl of cell suspension (1 × 10^6^ cells/ml) were activated with ConA (10 μg/ml) and a serial concentrations of rHcARF1 (5, 10, 20, and 40μg/ml). The control group was treated with ConA in equal volume of PBS. The plate was cultured at 37°C and 5% CO_2_ for 72 h. Then 10 μl of CCK-8 solutions (Beyotime Biotechnology, China) were added to each well of the plates 4 h before harvesting and the absorbance values at 450 nm (OD_450_) were measured using a microplate reader (Thermo Scientific, USA). The OD_450_ of controls were set as 100%. Cell proliferation index was calculated by the formula: OD_450_ rHcARF1 /OD_450_ control. Each experiment was performed in triplicate.

### Nitric oxide production assay

The goat PBMCs were harvested and washed twice with PBS. Then, 100 μl of cells (1 × 10^6^ cells/ml) were incubated either with PBS and a serial concentrations of rHcARF1 (5, 10, 20, and 40μg/ml) in 96-well plates in DMEM medium. Production of nitric oxide by PBMCs was determine by measurement of intracellular nitrite in the PBMC by using the Griess assay [65] according to the instruction of Total Nitric Oxide Assay Kit (Beyotime Biotechnology, China). Absorbance of the colored solution at 540 nm (OD540) in each well was measured using a plate reader (Bio-Rad Laboratories, USA). Absorbance values were converted to micromoles per liter (μmol/L) using a standard curve that was generated by addition of 0 to 80 μmol/L sodium nitrite to fresh culture media. Three individual experiments were performed.
